# Prospects for the treatment of drug-resistant malaria parasites

**DOI:** 10.2217/17460913.1.1.127

**Published:** 2006-06-26

**Authors:** Leann Tilley, Timothy ME Davis, Patrick G Bray

**Affiliations:** 1 Department of Biochemistry, La Trobe University, Melbourne, Victoria 3086, Australia. Tel.: +61 394 791 375; Fax: +61 394 792 467; L.Tilley@latrobe.edu.au; 2 School of Medicine and Pharmacology, University of Western Australia, Fremantle, Western Australia, Australia; 3 Molecular and Biochemical Parasitology Group, Liverpool School of Tropical Medicine, Pembroke Place, Liverpool, L3 5QA, UK

**Keywords:** antimalarials, artemisinin combination therapy, chloroquine, drug resistance, endoperoxide, malaria, PfCRT, quinolines

## Abstract

Widespread parasitic resistance has led to an urgent need for the development and implementation of new drugs for the treatment of *Plasmodium falciparum* malaria. Artemisinin and its derivatives are becoming increasingly important, used preferably in combination with a second antimalarial agent to increase the efficacy and slow the development of resistance. However, cost, production and pharmacological issues associated with artemisinin derivatives and potential partner drugs are hindering the implementation of combination therapies. This article reviews the molecular basis of the action of, and resistance to, different antimalarials and examines the prospects for the next generation of drugs to combat this potentially lethal human pathogen.

**Figure 1.**
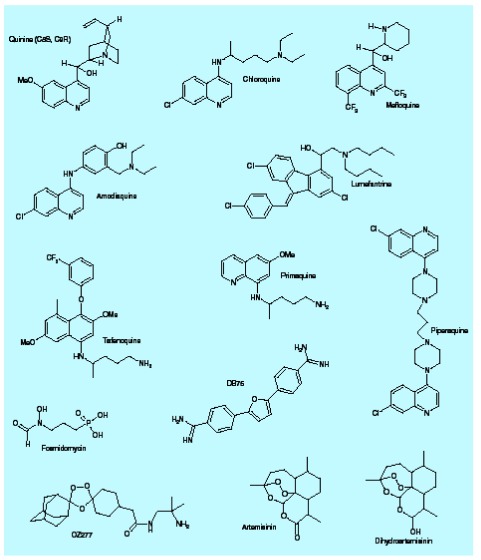
Structures of some antimalarial compounds.

**Figure 2.**
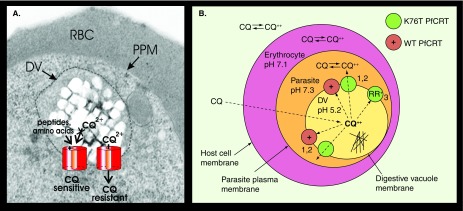
The role of PfCRT in CQ resistance. **(A)** Electron micrograph of the DV of a *Plasmodium falciparum*-infected erythrocyte, and illustration of the location and orientation of PfCRT at the DV membrane and its role in extrusion of CQ^2+^ in CQ-resistant parasites. The DV membrane is indicated by a broken grey line. **(B)** Diagram of CQ accumulation in drug-sensitive and -resistant parasites. CQ diffuses across cell membranes in its uncharged form and binds to two protons in the acidic DV. In CQ-sensitive (WT) parasites, the protonated form of CQ is impermeable to the DV membrane and CQ^2+^ accumulates in the DV. (1) Mutated PfCRT allows the transport of positively charged drugs. The PfCRT mutations that are responsible for CQ resistance make the transporter barrel more hydrophobic and the critical K76T mutation replaces lysine (a positively charged amino acid) with threonine (a neutral amino acid). (2) CQ is transported out of the DV along a large concentration gradient. The authors and others have proposed that charge loss in the CQ-resistant PfCRT mutants allows the transport of CQ^2+^ out of the DV away from its hematin target [26,146,147]. (3) Resistance reversers (RR^+^) bind in the pore of mutated PfCRT and block the efflux of CQ [146]. CQ: Chloroquine; DV: Digestive vacuole; PfCRT: *P. falciparum* CQ-resistance transporter; WT: Wild-type.

## Malaria: an intolerable burden

Every year hundreds of millions of new infections with *Plasmodium falciparum* malaria occur and up to 2 million children die annually from the disease [1,2]. Although most deaths occur in sub-Saharan Africa, malaria remains a significant health problem throughout most of South-East Asia, the Indian subcontinent, the South Pacific region and Latin America. With an effective vaccine still a distant prospect, chemotherapeutic measures are the mainstay of defense against malaria. The combination of a limited choice of effective antimalarials and the relentless development of parasitic drug resistance means that it is crucial that the right choices are made when new drugs are selected and that these drugs are introduced into the field in a way that prolongs their therapeutic life.

## Loss of the miracle drug, chloroquine

For 50 years, chloroquine (CQ) was the drug of choice for preventing and treating malaria because it is readily available, possesses appropriate pharmacological properties including a long half-life, is highly efficacious against sensitive parasites, and is safe and well tolerated when used in prescribed doses [3]. Most importantly, it is cheap. The cost of a course of CQ to treat a patient in Africa is approximately US$0.15. In countries where the total annual health expenditure per person may be lower than US$6 per year, this price enables access to treatment by even the poorest patients [4,5].

CQ has not always been used wisely and in an appropriate dose regimen. In the early 1960s, it was distributed as mass treatment in an effort to eradicate malaria. For example, in 1961, the WHO supplied 84,000 tons of CQ to Brazil alone for inclusion in table salt [6]. Shortly afterwards, the first cases of CQ-resistant *P. falciparum* were reported from the areas where the chloroquinized salt had been made available. CQ resistance has now reached all malaria-endemic regions of the world [7,8]. It retains some efficacy in semi-immune adults but much less in children who have not had enough time to develop immunity. Resistance to CQ is now also becoming a problem in the case of *Plasmodium vivax* [9,10]. The loss of CQ as an effective antimalarial has contributed to a doubling of malaria-related morbidity and mortality over the last two decades [11]. The effectiveness of CQ has now declined to the point where it is no longer the WHO-recommended treatment and has been officially abandoned in most countries. Nonetheless, newer drugs are too expensive for many and 300–500 million courses of CQ are still used each year [3,4,12,13].

CQ and other 4-aminoquinolines (Figure 1) accumulate in the parasite’s digestive vacuole, bind to hematin (ferriprotoporphyrin IX [FeIII-FP]) and inhibit its sequestration into hemozoin (Figure 2) [14–17]. Resistant parasites accumulate less CQ and exhibit less hematin binding, enabling them to survive in the presence of much higher concentrations of the drug [18–21]. Recent studies on CQ-resistant parasites have implicated specific polymorphisms in a protein referred to as PfCRT, the *P. falciparum* CQ-resistance transporter (Figure 2) [22,23]. PfCRT is a digestive vacuole membrane protein that may normally function as an amino acid transporter [22,24,25]. PfCRT alleles expressed by CQ-resistant parasites have fewer basic residues on the surface facing the digestive vacuole lumen (Figure 2). This may allow the leak of positively charged forms of CQ down an electrochemical gradient [26], and thereby increase the likelihood of parasite survival at plasma concentrations of CQ that would normally be therapeutic. Alternatively, PfCRT may function as an efflux pump [27]. Additive or compensatory mutations in other proteins may be needed for very high levels of CQ resistance [28]. PfCRT mutations appear to confer a fitness cost, as CQ resistance has been found to decline in areas where CQ pressure has been removed [3,29–32]. Unfortunately, this does not necessarily mean that acceptable efficacy is reinstated, especially in nonimmune patients [33].

## Development of quinoline-like replacements for chloroquine

Mutant *pfcrt* alleles appear to have arisen at multiple foci in South America, South-East Asia, Papua New Guinea and the Philippines [26,34]. From these foci, CQ-resistant parasites have gradually spread to all areas, reaching Africa in the late 1970s [35]. The inevitable demise of CQ as a useful therapeutic agent has triggered the development of a number of other quinoline antimalarials and aminoalcohols. These include the 4-aminoquinoline, amodiaquine, the bisquinoline, piperaquine, the quinoline methanol, mefloquine, the 9-phenanthrenemethanol, halofantrine, and the fluorenemethanol, lumefantrine (Figure 1, Table 1). Each of these compounds is more active than CQ against CQ-resistant parasites. This may be because they are more lipophilic and/or bulkier than CQ and therefore less effectively extruded by the mutant PfCRT [36]. Indeed, the aminoalcohols are more active against CQ-resistant parasites than against CQ-sensitive parasites, suggesting that they might bind preferentially to mutant PfCRT and block a critical transport function [37]. Resistance to these newer drugs is also increasing, especially for those, such as mefloquine, that have been used extensively [38,39]. Toxicity concerns have also limited the widespread use of halofantrine (a drug that is no longer recommended by the WHO because of cardiac effects), amodiaquine (as chemoprophylaxis rather than acute treatment), and, to a lesser extent, mefloquine [40]. Nonetheless, a number of these drugs are now being considered for use in combination with an artemisinin derivative [41].

Surprisingly little is known about the mechanism of action of these hydrophobic quinolines and aminoalcohols. Like CQ, they target blood-stage parasites and it has been suggested that they also target heme detoxification. This is probably true for amodiaquine and the bisquinolines, which are effective inhibitors of β-hematin formation [17,42–45]. However mefloquine, halofantrine, quinine and lumefantrine are relatively poor inhibitors of β-hematin formation *in vitro* [Klonis N, Tilley L, Unpublished Data] [17,45–49]. Thus, the molecular basis of their antimalarial activity is far from clear, and may involve targets additional to the hemoglobin degradation/heme detoxification pathway.

There is evidence for cross-resistance between CQ and other 4-aminoquinolines, but resistance to the aminoalcohols appears to involve a separate mechanism [8,16]. For example, relatively little resistance has developed to quinine, despite its use for over 300 years as a monotherapy [50]. This is likely to reflect the relatively short half-life of quinine (<1 day, compared with at least several weeks for the other quinolines) and consequently a limited opportunity for exposure of parasites to subtherapeutic plasma concentrations. Consistent with this hypothesis, clinically significant resistance to mefloquine (which has a half-life of approximately 2 weeks) developed within 6 years of its introduction in Thailand in 1984 [51,52]. The efficacy of quinine (which shares cross-resistance with mefloquine) has also started to decline in the same area [53]. Polymorphisms in the *P. falciparum* P-glycoprotein homolog-1 (Pgh1) have been shown to be associated with altered sensitivity to mefloquine, quinine and lumefantrine, as well as artesunate [38,54–56]. Resistance to mefloquine [38] and artemether–lumefantrine [57] has also been correlated with an increased copy number of the *pfmdr1* gene. Associations between polymorphisms in other ATP-binding cassette transporters (referred to as G7 and G49) and increased resistance to quinine, artesunate and dihydroartemisinin have also been noted [58].

## Resistance-reversing agents

One potentially useful strategy for overcoming drug resistance would be to use CQ in combination with a compound that reverses CQ resistance or acts synergistically. Indeed, a number of weakly basic amphipaths (such as verapamil, desipramine and chlorpromazine) that possess only very poor antimalarial activity themselves are able to potentiate the activity of CQ against CQ-resistant parasites [18,59–61]. Many of these compounds share certain structural features with CQ but are more lipophilic and less basic [62,63]. This has led to the suggestion that they act by binding to and blocking the mutated PfCRT pore [64].

Resistance-reversing agents have been considered for clinical use. For example, the antihistamine, chlorpheniramine, was shown to reverse CQ resistance in African field isolates [65] and a later clinical trial demonstrated enhanced efficacy of a CQ/chlorpheniramine combination over CQ alone for treating uncomplicated malaria in children [66], although the doses used were too large to be practical for wide-scale use. Recently, the authors’ group has shown that the 8-aminoquinolines, primaquine (PQ) and tafenoquine, are effective synergizers of the activity of CQ against resistant parasites [64]. PQ is already licensed for use in combination with CQ for treating the liver forms of *P. vivax* malaria. Compared with other suggested combinations, the low cost of a CQ–PQ combination is a major advantage; treatment with PQ costs approximately US$0.15. However, due to the serious toxicity (hemolysis) that can occur in patients with glucose-6-phosphate dehydrogenase (G6PD) deficiency, the patient’s G6PD status would need to be assessed before treatment with a CQ–PQ combination. Thus, PQ cannot be used in unregulated settings and this may limit its widespread application. A simple and inexpensive test is available [67], or alternatively, other similar compounds that are less toxic might be suitable as inexpensive synergizers of CQ action [64].

Despite generating a large volume of preclinical research over nearly two decades, resistance-reversing agents are yet to establish a place in the clinical management of patients with malaria. Issues of cost, compliance, efficacy and/or side effects have proved major impediments but, because of the ever-present specter of parasitic drug resistance, they should not be discarded.

## Novel 4-aminoquinolines & related drugs

Amodiaquine (Figure 1) is effective against many CQ-resistant strains of *P. falciparum.* However, clinical use of this 4-aminoquinoline has been severely restricted due to reports of hepatotoxicity and agranulocytosis when the drug is used for prophylaxis [68]. The amodiaquine side-chain contains a 4-aminophenol group capable of forming potentially toxic quinoneimine metabolites [69,70]. Structure–activity studies suggest that the presence of a 4-arylamino moiety provides analogs with superior activity against CQ-resistant strains and that the presence of an aromatic hydroxyl function is important for additional levels of antiparasitic activity. Thus, the interchange of the 3´-Mannich side chain with the 4´-hydroxyl function should provide a new template for active new drugs that are chemically incapable of forming potentially toxic quinoneimine metabolites. Isoquine, the direct isomer of amodiaquine, has emerged as the lead candidate. This compound has potent activity against CQ-resistant parasites *in vitro* and oral activity in rodent models of malaria, and should be as cheap to prepare as amodiaquine on an industrial scale [71]. Isoquine is currently in preclinical evaluation in a partnership between the Medicines for Malaria Venture (MMV) and GlaxoSmithKline (GSK) Pharmaceuticals [71].

Piperaquine (Figure 1) is a bisquinoline antimalarial drug that was first synthesized in the 1960s, and was used extensively in China and Indochina as prophylaxis and treatment over the next 20 years [41]. Piperaquine is thought to have a similar mode of action to CQ and other 4-aminoquinolines. It has comparable activity to CQ against CQ-sensitive strains of *P. falciparum* and *P. vivax* and better activity against CQ-resistant strains. With the development of piperaquine-resistant strains of *P. falciparum* and the emergence of the artemisinin derivatives, its use declined during the 1980s. However, during the next decade, piperaquine was rediscovered by Chinese scientists as one of a number of compounds suitable for use in combination with an artemisinin derivative [41].

## Advent of artemisinin & derivatives as front-line antimalarials

The ever-increasing level of resistance to the quinoline and antifolate antimalarials [72], has been offset, to some extent, by the discovery of the artemisinins. In 1971, Chinese scientists demonstrated antimalarial activity in extracts of a species of sweet wormwood, *Artemesia annua* (known locally as qinghao), and identified the active ingredient as qinhaosu or artemisinin [73–76]. Since then, a number of more easily formulated derivatives, such as artemether, artesunate, arteether and dihydroartemisinin have been developed for clinical use (Table 1).

Artemisinin is a sesquiterpene lactone with a 1,2,4-trioxane heterocycle (Figure 1). The endoperoxide moiety is essential for activity [77]. The precise mode of action of artemisinin-related compounds is still a matter of debate and the active endoperoxides are known to accumulate in a variety of parasite compartments, including the cytosol, digestive vacuole and membranes. They interact with ferrous iron (FeII) or with the reduced form of heme (ferroprotoporphyrin IX [FeII-FP]). FeII-FP is thought to activate the endoperoxide moiety to form reactive oxygen species and a carbon-centered radical intermediate that reacts with susceptible groups within parasite enzymes [77–82]. Artemisinins also form covalent adducts with hemozoin in the digestive vacuole [77,83,84]. FeIII-FP is formed in the digestive vacuole during hemoglobin digestion. Due to the oxidizing environment there can only be a tiny proportion of FeII-FP in the digestive vacuole [85]. This may be enough to activate endoperoxides by a catalytic mechanism and thereby inhibit targets in this compartment [86,87]. Alternatively, a small proportion of FeIII-FP may leak out of the digestive vacuole into the reducing environment of the parasite cytosol and be converted into FeII-FP [88,89]. This mechanism would allow activated endoperoxides to target proteins in the parasite cytosol.

The activity of artemisinin against cultured *P. falciparum* is enhanced by increased oxygen tension and by pro-oxidant compounds [90,91], indicating that activated oxygen may be an important mediator of the antimalarial activity of endoperoxide antimalarials. However, artemisinin is active against parasites maintained in a carbon monoxide atmosphere [91], indicating that alternative inhibitory pathways are also contributory [92]. Treatment of parasites with artemisinin results in morphological changes to various organelles, including the mitochondria, rough endoplasmic reticulum and nuclear envelope [93], although these changes can also occur after exposure to other classes of antimalarial drugs. Proteomic studies indicate that exposure of *P. falciparum* to artemether leads to upregulation of a range of glycolytic enzymes and stress response proteins [94]. Given the remarkable potency of endoperoxide antimalarials, it is likely that the lethal effects of the drugs are due to free radical-induced damage of particular proteins rather than more general damage caused by rapidly diffusing reactive oxygen species. Indeed, recent studies have provided convincing evidence that the *P. falciparum* homolog of the sarco/endoplasmic reticulum Ca^2+^-ATPase (also known as PfATPase6) is a major downstream target of activated artemisinin in the parasite cytosol [95,96]. Other targets, such as digestive vacuole enzymes and the translationally controlled tumor protein homolog, have also been implicated [86,87]. Components of the mitochondrial electron transport chain have been shown to be susceptible to artemisinin in yeast [97]; however, it is not clear if these are important targets in plasmodia.

Clinically significant artemisinin resistance has not yet been seen in the field. However, decreased susceptibility to artemisinin is readily observed in laboratory strains [77,98–100]. It can be associated with the same polymorphism in Pgh1 that confers resistance to mefloquine [38,56]. Mutations in a key residue (L263E) in PfATPase6 result in decreased susceptibility to artemisinin in an oocyte expression model [96]. There is no association between this particular polymorphism of PfATPase 6 and artemisinin sensitivity in field isolates [38]; however, a reduced response to the related compound, artemether, shows association with another polymorphism (S769N) of PfATPase6 in some field isolates [101]. Polymorphisms in PfCRT also affect sensitivity to mefloquine, halofantrine and artemisinin [102,103]. *Plasmodium berghei* can develop stable resistance to artemisinin without mutations in PfATPase6, PfCRT or Pgh1 [99], indicating that other genes may also be involved. It is critical that a better understanding of artemisinin action and resistance is obtained so that strategies can be developed to retard the development of resistance in the field.

## Problems with the implementation of artemisinin combination therapies

Whatever the mechanism of action, artemisinin-related drugs are extremely fast-acting and highly potent, making them suitable for treating both severe and uncomplicated malaria [104,105]. However, artemisinin and its derivatives are not suitable for prophylaxis or for use as monotherapy due to their very short half-lives *in vivo* (from minutes to hours). Even with a 5-day treatment regimen, recrudescences are frequently observed if these compounds are used alone [106]. To ensure cure and to encourage compliance, patients are usually treated with a 3-day dose regimen comprising an artemisinin-related compound in combination with a second drug with a longer half-life [104]. Since 2001, the WHO has recommended that uncomplicated malaria should be treated with a combination of an artemisinin derivative and a drug with a different mode of action in areas where *P. falciparum* is predominant. Experience with mefloquine and artesunate in South-East Asia has led some to argue that combinations with complementary pharmacokinetics (i.e., a short half-life artemisinin and a long half-life partner drug) are most effective. This is despite concerns that mismatched pharmacokinetics allow parasites to evolve resistance sequentially as the longer half-life partner persists in a vulnerable time-window, a result that could undermine the benefits of artemisinin combination therapies (ACTs) [107].

Artemisinin combinations are more effective than conventional treatments in regions where drug resistance levels are high; however, cost is a major issue for wider implementation [108–112]. Artemisinin combinations currently cost at least US$1.20 per adult treatment course, much more expensive than CQ or sulphadoxine/pyrimethamine and too costly for many patients [4,5,109]. In Africa, only approximately 50% of children with fever in malarious areas receive any treatment and 84% of these are treated with CQ, which is largely ineffective [113]. In an effort to enable the implementation of ACTs in Africa, there have been calls for international donors to cover the increased cost. The Global Fund to Fight AIDS, Tuberculosis and Malaria has received US$3.7 billion from donors to help fund programs for 2006/2007; however, this represents only approximately half of the amount required [4,114,115].

Interestingly, in areas of high endemicity, less expensive combinations can remain more effective than ACTs [116–122]. Yeung and colleagues are generating a model to predict the cost–effectiveness of introducing ACT under different implementation scenarios [51]. The model may help policy makers, as it takes into account parasite factors (such as transmission rates and levels of drug resistance), host factors (such as immunity) and economic factors.

The greatly increased demand for ACT has led to problems with the supply of formulations prepared according to Good Manufacturing Practice (GMP) [74]. Artemisinin is prepared by large-scale extraction from plants and its derivatives are generated semi-synthetically from the purified extract. A period of over 1 year is needed for the horticultural, harvesting, extraction and manufacturing processes [109,110]. Plants from different areas vary in the amount of artemisinin they produce. High-yielding plants come mainly from plantations in northern Vietnam and in China and the best plants grow at altitudes above 1000 m. This greatly limits the ease of scale-up of the process. Nevertheless, locally manufactured non-GMP artemisinin combinations are increasingly available in a number of South-East Asian countries, and Chinese ACT formulations are now exported widely.

The supply of counterfeit or inferior drugs is a major problem in the treatment of malaria [123] and the WHO has instituted a qualification process for artemisinin combinations in an effort to limit the distribution of fake drugs. However, artemether–lumefantrine (Coartem^®^) is currently the only GMP-registered ACT. Coartem is produced as a nonprofit collaboration between Novartis and Kunming Pharmaceuticals. A total of 100 million treatments will be generated in 2006, representing approximately 50-times more than the company predicted would be needed at this stage [115]. The Coartem combination is not ideal; lumefantrine is an expensive partner drug and the six-dose, 3-day regimen requires the drug to be taken with food to improve absorption.

Piperaquine (Figure 1) has been combined with dihydroartemisinin with a view to providing a well-tolerated, short-course treatment regimen with a high cure rate against drug-resistant parasites. This combination has been used in South-East Asia and clinical trials have demonstrated good efficacy against CQ-resistant parasites [44,124,125]. One available formulation ‘duo-cotecxin’ (Holleykin Pharmaceutical Company, China) is cheaper than Coartem (∼US$1.20 per adult treatment compared with US$2.40). However, it is not yet registered nor manufactured to GMP standards [115]. An MMV project has been introduced to help piperaquine–dihydroartemisinin to meet international standards and a comprehensive clinical trial program has begun in Africa and South-East Asia. One issue of concern is that piperaquine has a mean elimination half-life of 23 days in adults [126] and very low plasma concentrations may persist for several months [127]. This profile is dramatically different to that of dihydroartemisinin (half-life: ∼1 h). Thus, the dihydroartemisinin combination will be subject to the same concerns outlined above for other ACT combinations.

Coformulated combinations of artesunate with amodiaquine or mefloquine are also under development by Sanofi–Aventis in partnership with the Drugs for Neglected Diseases Initiative (DNDi), while the MMV is working with industrial partners to develop a LapDap^™^ combination (chlorproguanil–dapsone–artenusate) and a pyronaridine–artenusate combination (see Figure 1 for structures of some of these compounds).

Biotechnology approaches are being explored with the aim of generating artemisinin precursors in bacteria and yeast at low cost and in large quantity [115,128,129]. A final price of US$1 per GMP ACT is being targeted, although even at this price the drug may still need to be heavily subsidized by donor nations to be affordable [4]. In the meantime, there is a risk that lower quality ACT formulations will be employed, which may accelerate the development of resistance [115]. Most African countries have made policy decisions to implement ACTs as the preferred antimalarial therapy with little hope of being able to source the drugs or to afford them [98,109,130].

In addition, there is a lingering concern regarding artemisinin neurotoxicity [98]. Brain stem lesions can occur in animals receiving high doses over long periods of time, but there have been no convincing cases of neurological involvement in humans treated with artemisinin drugs, including those who have received repeated courses. Artemisinins have also been shown to cause embryotoxicity in some animals, although there is no evidence to date for human fetal toxicity [98,131]. The WHO currently recommends that artemisinin is contra-indicated for pregnant women in the first trimester – one of the major groups requiring prompt, effective antimalarial treatment – and should be used with caution in later pregnancy [98,132].

## Novel endoperoxides

A number of medicinal chemistry groups have attempted to improve the pharmacokinetic properties of the artemisinins and overcome production problems. Due to limited space, this discussion will be restricted to those compounds already in development. An important consideration in the design of any new peroxide analog is the concern of neurotoxicity. Any analog with a higher logP than artemether is likely to cross the blood–brain barrier [74]. Haynes and coworkers have prepared new analogs with potentially reduced neurotoxicity by applying the ADME paradigm (used for optimizing absorption, distribution, metabolism and excretion). This has resulted in analogs with enhanced water solubility, which increases gastrointestinal absorption but reduces passage across the blood–brain barrier [133]. Artemisone (a Bayer product) is one such analog that illustrates the success of the ADME approach to drug design.

Other groups have taken a totally synthetic route in the development of novel endoperoxides. Organic chemists have long known that exposing simple alkenes to ozone creates an endoperoxide bridge in the resulting ozonide that is identical to the critical endoperoxide bridge of artemisinin. However, the resulting ozonides are chemically unstable, making them useless as potential antimalarials. In a key breakthrough, Vennerstrom and colleagues showed that stability could be dramatically improved by substituting a bulky adamantane cage onto the ozonide [134]. Some of these compounds exhibited better activity even than artemisinin itself, but still suffered from poor water solubility. This problem was subsequently solved and MMV is currently supporting the development and testing of one of the second generation compounds, known as OZ277 (Figure 1). This compound has a longer half-life than artemisinin, appears to be well absorbed, has an acceptable toxicity profile and is suitable for industrial level scale-up of synthesis [134,135]. It has been shown to be highly active against field isolates from Gabon [136]. A product is expected to reach the market by 2009, with a target price of US$1 per treatment.

## Public–private partnerships for the development of novel antimalarials

The development of new antimalarial drugs and combinations is currently receiving a boost from a number of public–private partnerships. MMV was developed in 1999 by the WHO, the World Bank, donor governments, philanthropic institutions and industrial partners. The MMV encourages and brokers partnerships between public and private institutions to develop existing candidate drugs and to produce new leads [8]. The Gates Foundation currently provides approximately 60% of the US$30 million annual budget used to manage its approximately 25 projects [12,115]. For example, GSK has a research facility in Tres Cantos, Spain, working on diseases of the developing world. GSK employs 25 scientists at this site who are matched by 25 scientists provided by MMV. Similarly, Sanofi–Aventis is working closely with the DNDi [115]. MMV expects to have one to two novel combination antimalarials available on the market by 2007 and possibly three to four more by 2010.

A number of antibiotics, such as azithromycin, clindamycin and the tetracyclines are being developed and tested for use in combination with quinine and other drugs [13,98]. Clindamycin–fosmidomycin (Figure 1) is a new combination that targets pathways in the parasite’s apicoplast with good efficacy [137,138]. Sanofi–Aventis are developing ferroquine, trioxaquine, thiazolium and choline-uptake inhibitors. MMV projects include a prodrug of the diamidine, DB75 (Figure 1) [139], inhibitors of the fatty acid biosynthetic enzyme, Fab I, and the cysteine protease, falcipain, and a 4(H)-pyridone derivative. Additional projects include the development of inhibitors of anion channels and farnesyl transferases [140–142], as well as a novel aminoquinoline (AQ13) [143].

## Conclusions

Artemisinin is already being used as a first-line treatment in many countries in South-East Asia and Africa. Delaying the implementation of effective ACTs in regions of high drug resistance will encourage the unregulated use of the artemisinins and partner drugs as monotherapies, which could further exacerbate the problem of drug resistance. However, the choice of which combination to use remains controversial. An ideal drug combination would contain two or more drugs with different modes of action. Each drug would be safe, well tolerated, effective and readily available in fixed dose combination, preferably as a single dose or at most as a 3-day regimen. At least one of the drugs should be fast-acting and exhibit a broad spectrum of action. Most importantly, the drugs must be affordable. Recent reviews provide an overview of the currently available drug combinations, making it clear that the ideal combination does not currently exist [72,98]. Important challenges for the immediate future include the registration of additional artemisinin combinations with suitable inexpensive partner drugs. In the longer term it is imperative that cheap synthetic endoperoxides with longer half-lives are developed and matched with partners with similar pharmacokinetic profiles. It may be necessary to employ triple combinations to ensure that at least two drugs are present in the patient’s system long enough to ensure cure [144]. Alternatively, different effective treatment regimens can be rotated within a country or region to safeguard against resistance, with changes directed by regular *in vivo* and *in vitro* monitoring of efficacy. Clearly, any new antimalarial therapies need to be integrated into other strategies aimed at controlling disease, such as mosquito control, impregnated bed nets, improved management of severe malaria and intermittent preventative treatment regimens for pregnant women. It now appears likely that the aim of the Roll Back Malaria campaign, to half malaria deaths by 2010 [4,114,145], will not be achieved. If a malaria disaster is to be averted there are many challenges to be faced in the coming decade.

## Future perspective

The development of novel antimalarials is an expensive and slow process and the implementation of new drug policies requires resources not readily available in the third world. Nonetheless, a recent upswing in political will and in public and private funding of efforts to tackle the problem of malaria ensures that any effective (and inexpensive) new antimalarials will progress rapidly through the drug development pipeline and will be translated into the field. The bitter experience of watching the inexorable development of resistance to each of the antimalarials that was developed in the 20th century tells us that it is critical that we make appropriate use of the handful of effective drugs currently in our armory or now under development. A global approach to the coordinated and rapid deployment of ACTs is essential for malarious regions with entrenched resistance to other antimalarial drugs. It is critical that decisions regarding which drug regimen to change to, and how to implement the changes, are made in a way that maximizes the benefit to the patient while minimizing the risk of the development of drug resistance. Finally, it is essential that any policy decisions to implement more expensive antimalarials are funded by substantially increased inputs from donor nations.

**Table 1.** Properties and status of some current drugs and some drugs under development.

Drug	Elimination half-life	Efficacy	Advantages/ disadvantages	Status
Chloroquine	3–14 days	Major resistance; some efficacy in partially immune patients	Cheap Readily available	Not recommended Still widely used for treatment
Sulphadoxine/ pyrimethamine (Fansidar^®^)	6–9 day for sulphadoxine; 3–4 days for pyrimethamine	Major resistance; some efficacy in partially immune patients	Cheap Readily available	Only recommended in areas of low-grade resistance Still widely used for treatment
Mefloquine	2–3 weeks	High, except in some regions of South-East Asia	Can produce neurological side effects Expensive	Has been used in ACT
Quinine	10–20 h	High	Tinnitus, giddiness, hypoglycemia Hemolysis in G6PD-deficient patients	Use limited by toxicity and compliance issues Used to treat severe malaria
Malarone	12–15 h for proguanil; 2–3 days for atovaquone	Good prophylaxis efficacy Few studies of efficacy as treatment	Expensive	Use limited by cost and potential for development of resistance
Lumefantrine	4–6 days	High	Expensive Must be taken with food	Component (with artemether) of the only GMP-registered ACT now available
Piperaquine	3–4 weeks	High	Inexpensive Increasingly available	Combination with dihydroartemisinin under development
Isoquine	Under investigation Slowly eliminated active metabolites	High, even against chloroquine-resistant strains	Readily synthesized. No amodoaquine-like side effects	Under development by MMV/GSK
Artemether	∼1 h	High Rapid action Problems with recrudescence	Oil-soluble Can be used orally or as an i.m. injection	Used in combination with mefloquine in South-East Asia
Dihydroartemisinin	∼1 h	High Rapid action Problems with recrudescence	Oil-soluble Can be used orally or rectally	Combinations with piperaquine under development
Artesunate	∼1 h	High Rapid action Problems with recrudescence	Water soluble Fastest acting artemisinin derivative Can be used parenterally, rectally or orally	Combinations with pyronaridine, amodiaquine, mefloquine and chlorproguanil–dapsone under development
Artemisone	∼1 h	High	Increased water solubility Potential neurological side effects less likely than other artemisinin drugs	Some stability problems Development under review
OZ277	∼2 h	High	Fully synthetic	Trials underway
Fosmidomycin	1–5 h	Early trials are promising	Targets a novel pathway in the apicoplast	Under development in combination with clindamycin
Clindamycin	2–4 h	Moderate to high	Inhibits protein synthesis	Under development in combination with fosmidomycin

ACT: Artemisinin combination therapy; G6PD: Glucose-6-phosphate dehydrogenase; GMP: Good manufacturing practice; GSK: GlaxoSmithKline; i.m.: Intramuscular; MMV: Medicines for Malaria Venture.

**Executive summary**

**Antimalarial drug resistance: a major problem**

• Each year, *Plasmodium falciparum* causes hundreds of millions of malaria infections and up to 2 million deaths, particularly of children in sub-Saharan Africa.
• The mainstay antimalarials, chloroquine (CQ) and sulphadoxine/pyrimethamine, are now largely useless due to the development of drug resistance. Resistance to other quinoline antimalarials is also increasing. This has led to a critical need to develop novel drugs or drug combinations that can circumvent resistance.
• Artemisinin and its derivatives are becoming increasingly important in the treatment of drug-resistant malaria.

**Mechanism of resistance to chloroquine & other quinolines**

• The molecular basis of *P. falciparum* resistance to the quinoline antimalarials is incompletely understood, although recent studies have detailed the importance of a parasite protein known as the *P. falciparum* CQ resistance transporter (PfCRT).
• A critical PfCRT mutation (K76T) allows the transport of positively charged CQ away from its site of action in the parasite’s digestive vacuole. Polymorphisms in another digestive vacuole-located protein, *P. falciparum* P-glycoprotein homolog-1 (a homolog of the human multidrug resistance protein) are thought to be responsible for altered sensitivity to other quinolines, such as mefloquine, quinine and lumefantrine, and can be associated with decreased sensitivity to artemisinin derivatives.
• Because the mechanisms of resistance involve alterations in drug accumulation rather than changes in the drug target, it remains possible that novel quinolines will be developed (possibly used in conjunction with synergizers) that will circumvent the resistance mechanisms.

**Artemisinin & derivatives as front-line antimalarials**

• Developed by Chinese scientists from a traditional antimalarial treatment, artemisinin, and its more easily formulated derivatives, are now the major hope for treating drug-resistant malaria.
• These endoperoxide antimalarials are activated by reduced heme or iron and induce free radical-mediated damage to parasite proteins.
• The *P. falciparum* homolog of the sarco/endoplasmic reticulum Ca^2+^-ATPase (also known as PfATPase6) appears to be a major target of artemisinin.

**Artemisinin combination therapy: pharmakinetic issues**

• Artemisinin and its derivatives are not suitable for prophylaxis or for use as monotherapies due to their very short half-lives *in vivo* (from minutes to hours). The WHO has recommended a 3-day dose regimen in combination with a second drug with a longer half-life. This is known as artemisinin combination therapy (ACT). However, there are major concerns that mismatched pharmacokinetics will allow parasites to evolve resistance to the longer half-life partner, undermining the benefits of ACTs.
• Clinically significant artemisinin resistance has not yet been seen in the field; however, decreased susceptibility has been observed in laboratory strains and field isolates. It is critical that a better understanding of artemisinin resistance is obtained so that strategies can be developed to retard the development of resistance in the field.

**Cost & production issues**

• The greatly increased recent demand for ACTs has led to problems with the supply of formulations prepared according to Good Manufacturing Practice (GMP). This has led to the supply of lower quality or fake ACT formulations, which may accelerate the development of resistance. Moreover, cost remains a major issue preventing the wider implementation of ACTs. ACTs cost US$1.20–2.40 per adult treatment course, much more expensive than CQ or sulphadoxine/pyrimethamine (∼US$0.15).
• With improved production practices, a final price of US$1 per GMP ACT is being targeted; however, even at this price the drugs will still need to be heavily subsidized to be affordable. Many African countries have made policy decisions to implement ACTs with little hope of sourcing or affording the drugs. In an effort to enable the implementation of ACTs in Africa, there have been calls for international donors to cover the increased cost.

**Public-private partnerships: the way forward for new antimalarials**

• A recent upswing in political will and in public and private funding has enabled the development of a number of public–private partnerships (fostered by institutions such as the Medicines for Malaria Venture) for the development of antimalarial drugs. This ensures that new antimalarials progress rapidly through a drug development pipeline and are translated into the field.

**Conclusion**

• A global approach to the deployment of ACTs is needed to enable effective ACTs to be utilized in regions of high drug resistance in the immediate future. ACTs need to be implemented in a manner that maximizes the benefit to the patient while minimizing the risk of the development of drug resistance. The drugs employed in any ACT need to be safe, well tolerated, effective and readily available in a fixed-dose, short-course regimen. Most importantly, the drugs must be affordable.
• Tackling the problem of drug-resistant malaria represents a major challenge that must be faced in the next decade.
